# Using Stakeholder Perspectives to Improve End-of-Life Training Opportunities

**DOI:** 10.1093/geroni/igaf122.1417

**Published:** 2025-12-31

**Authors:** Britteny Howell, Raven Weaver, Tina Newsham

## Abstract

Health and human services professionals are often underprepared to support patients with end-of-life (EOL) decision-making and supporting the bereaved, largely due to gaps in their professional training. Colleges and universities can fill this gap by providing courses to increase the knowledge of the future workforce around issues related to death and dying, but faculty teaching these courses may themselves also be underprepared for the realities of teaching this challenging material. In this symposium, presenters from several different fields and universities will address two goals: (1) fill some remaining knowledge gaps regarding how to teach about advance care planning (ACP) and EOL discussions and (2) suggest resources and self-care strategies for those teaching courses on death and dying. The session will begin with three presentations presenting process data and stakeholder perspectives on death education. The first paper will present preliminary process data on designing and implementing a pilot program where medical and health professional students facilitate ACP discussion events in rural communities. The next presentation will discuss data collected from a study on faculty perspectives of the benefits and challenges of teaching and talking about death and dying. Lastly, the third paper will present medical students’ and older adult patient’s perspectives regarding the need for EOL training and teaching strategies. The session will wrap up with a final presentation from a multidisciplinary team who will provide some personal benefits and self-care strategies from those who teach death and dying courses.

